# An Artificial Intelligence-Based Tool for Enhancing Pectoral Muscle Segmentation in Mammograms: Addressing Class Imbalance and Validation Challenges in Automated Breast Cancer Diagnosis

**DOI:** 10.3390/diagnostics14192144

**Published:** 2024-09-26

**Authors:** Fausto David Cortes-Rojas, Yazmín Mariela Hernández-Rodríguez, Rafael Bayareh-Mancilla, Oscar Eduardo Cigarroa-Mayorga

**Affiliations:** 1 Departamento de Ingeniería Eléctrica/Sección de Bioelectrónica, Centro de Investigación y de Estudios Avanzados del IPN, Av. Instituto Politécnico Nacional 2508, Col. San Pedro Zacatenco, Gustavo A. Madero, Ciudad de México 07360, Mexico; fdavid.cortezr@cinvestav.mx; 2 Departamento de Tecnologías Avanzadas, UPIITA-Instituto Politécnico Nacional, Av. IPN No. 2580, Ciudad de México 07340, Mexico; yazmin.hernandez@cinvestav.mx

**Keywords:** convolutional neural networks, class imbalance function, breast tissue classification, breast cancer

## Abstract

Breast cancer remains a major health concern worldwide, requiring the advancement of early detection methods to improve prognosis and treatment outcomes. In this sense, mammography is regarded as the gold standard in breast cancer screening and early detection. However, in a scenario where extensive analysis is required, a large set of mammograms conducted by radiologists may carry out false negative or false positive diagnoses. Therefore, artificial intelligence has emerged in recent years as a method for enhancing timing in breast cancer diagnosis. Nonetheless, preprocessing stages are required to prepare the mammography dataset to enhance learning models to correctly identify breast anomalies. In this paper, we introduce a novel method employing convolutional neural networks (CNNs) to segment the pectoral muscle in 1288 mediolateral oblique mammograms (MLOs), thereby addressing class imbalance and overfitting between classes, and dataset augmentation based on translation, rotation, and scale transformation. The effectiveness of the model was assessed through a confusion matrix and performance metrics, highlighting an average Dice coefficient of 0.98 and a Jaccard index of 0.96. The outcomes demonstrate the model capability to accurately identify three classes: pectoral muscle, breast, and background. This study emphasizes the importance of tackling class imbalance problems and augmenting data for the training of models for reliable early breast cancer detection.

## 1. Introduction

Breast cancer remains one of the most significant global health challenges of our time, being the most commonly occurring cancer in women worldwide and the most common cancer overall. In 2022, an estimated 287,850 new cases of invasive breast cancer and 51,400 cases of ductal carcinoma in situ (DCIS) are expected to be diagnosed in U.S. women, with 43,250 anticipated deaths. Breast cancer incidence has increased by 0.5% annually in recent years, largely due to localized-stage and hormone receptor-positive disease, while mortality rates have declined by 43% from 1989 to 2020, with 460,000 lives being saved. However, racial disparities persist, with Black women experiencing a 40% higher death rate compared to White women, underscoring the need for equitable access to screening and treatment [1,2]. The disease’s pervasiveness and the toll it takes on individuals and families underscore the urgent need for innovative and effective methods for early diagnosis and treatment.

Despite the statistics, the global healthcare community has made significant strides in breast cancer treatment and early detection, leading to improved survival rates. For instance, the 5-year relative survival rate for women in the United States with non-metastatic invasive breast cancer is 91%, and the survival rates continue to improve with advancements in medical research and technologies [3]. These advancements underscore the importance of continued investment in the research and development of more precise, effective diagnostic tools and treatments, particularly in the field of early diagnosis where the potential to significantly impact survival rates is immense.

Approximately 30% of breast cancer incidences are linked to lifestyle choices that can be altered, such as maintaining a healthy weight, engaging in regular physical activity, and moderating alcohol consumption. These factors suggest a significant portion of breast cancer cases might be preventable through changes in personal health practices. Additionally, the role of mammography screening as a form of secondary prevention cannot be overstated, as it plays a crucial role in early detection, thereby reducing the likelihood of death from breast cancer. Coupled with the ongoing improvements in medical treatments, these preventive measures have been instrumental in the notable decrease in breast cancer mortality rates [1,4,5].

Research and the implementation of methods for early diagnosis have experienced significant growth in recent decades, particularly in the application and development of technologies involving artificial intelligence (AI). Particularly with the application of convolutional neural networks (CNNs), medicine has been beneficial through several fields. These methods, rooted in deep learning (DL), are remarkable at analyzing medical images, which may offer a powerful tool for medical diagnosis. The growth in the popularity of CNNs in the medical field, especially for breast cancer diagnosis through mammogram analysis, is a testament to the significant advancements and potential benefits AI brings to healthcare [6,7]. CNNs are a specialized category within DL networks, engineered to learn spatial hierarchies of features directly, autonomously, and adaptively from images. The foundation of their capability lies in the convolutional layers, which employ a series of filters on the input images to generate feature maps. These maps effectively summarize the detected features within the images. The architecture is further enhanced by pooling layers, which serve to reduce the dimensionality of the feature representation, and fully connected layers, which are instrumental in making decisions based on the distilled features. This intricate combination of processing layers renders CNNs extraordinarily effective for tasks related to image recognition, setting them apart in the field of computer vision [8]. Medical imaging, which includes X-rays, MRI, and mammograms, generates vast amounts of data that require expert analysis. The traditional process is time-consuming and subject to human error, making AI a promising alternative. CNNs, with their ability to detect patterns and anomalies that may not be visible to the human eye, offer a powerful tool for enhancing diagnosis accuracy and efficiency. Breast cancer is one of the leading causes of cancer death among women worldwide. Early diagnosis is crucial for effective treatment and improved survival rates. Mammography is the most common screening method for breast cancer, but interpreting these images can be challenging and subjective. CNNs have emerged as a transformative solution in this area. Several studies have demonstrated the potential of CNNs to accurately classify mammographic images, distinguishing between benign and malignant lesions with high precision. This capability can significantly aid radiologists in making more accurate diagnoses, reducing the rate of false positives and false negatives. CNNs can analyze mammograms to detect subtle signs of breast cancer at an earlier stage than might be possible through traditional methods [9,10,11,12].

Although CNNs have demonstrated remarkable capabilities in enhancing the accuracy and efficiency of diagnosing based on medical images, the application of CNNs in mammography analysis still have challenges that may affect the diagnostic outcomes, leading to variances between false positives, false negatives, and accurate diagnoses. One of the primary challenges involves the presence of extraneous elements within mammograms, such as medical annotations and artifacts, which can introduce noise and interfere with the analysis. These non-target features can obscure or mimic the characteristics of pathological findings, complicating CNNs’ ability to accurately identify and classify lesions [13,14]. Another notable challenge is the reliance on two-dimensional projections of mammograms rather than image reconstructions that enhance the contrast within tissues. This limitation can lead to the loss of valuable spatial information about the structure and distribution of breast tissues, potentially affecting the detection and characterization of abnormalities [15,16]. Moreover, the presence of other tissues, notably the pectoral muscle, poses additional difficulties. The segmentation of the pectoral muscle in mammographic analysis plays a crucial role in enhancing the clarity and focus of diagnostic assessments. By accurately isolating and removing this non-breast tissue, our methodology significantly reduces interference in the visual field, which is particularly beneficial for the detailed examination of subtle oncological markers such as microcalcifications and tissue distortions. These markers are crucial for early breast cancer detection but can be easily obscured by overlapping muscular structures. Our approach not only minimizes the likelihood of false positives, which are often a result of misclassifying muscle tissues as suspicious or malignant, but also improves the specificity and sensitivity of the detection algorithms. This refined focus on relevant breast tissues ensures that each mammogram is assessed with heightened accuracy, facilitating the detection of early-stage breast cancer, which is paramount for effective treatment and improved patient outcomes. Furthermore, the standardized exclusion of the pectoral muscle enhances the consistency across imaging studies, essential for reliable longitudinal studies and multi-site collaborations, where uniform data quality is critical for the aggregation and comparison of findings [17,18,19]. Variations in tissue density and intensity across different regions of the mammogram can result in discrepancies in the interpretation of the images. Innovations in mammographic image analysis have centered on enhancing diagnostic accuracy, particularly through the segmentation of anatomical features such as the pectoral muscle. Existing methods often focus on general segmentation techniques that do not differentiate between closely packed tissues effectively [20,21,22,23,24,25]. Our method introduces a novel approach by incorporating advanced machine learning algorithms that specifically target the pectoral muscle with enhanced precision. This technique leverages deep convolutional neural networks (CNNs) optimized through adaptive learning rates and class-specific weighting, addressing the common challenges of over-segmentation and under-representation of minor but critical features. Such variations can particularly impact the detection of masses or microcalcifications, as the CNN might struggle to distinguish these pathological features from normal anatomical structures. Furthermore, the diversity in mammographic appearances due to factors such as patient age, breast density, and the stage of disease complicates the task of developing a universally effective CNN model. The variability in image quality and the need for large, annotated datasets for training CNNs also represent significant hurdles in achieving optimal performance [26].

To address these challenges, ongoing research and development are focusing on improving CNN architectures, data preprocessing techniques, and training methodologies. Efforts are being made to enhance the robustness of CNNs against artifacts and non-target features and develop strategies for effectively managing variations in tissue appearances. Additionally, the integration of CNNs with other diagnostic modalities and clinical data is being explored to provide a more comprehensive and accurate assessment of mammographic images. Table 1 offers a comprehensive summary of the current advancements in research concerning pectoral muscle segmentation. This table offers a comprehensive summary and review of diverse methodologies, all aimed towards the common goal of segmenting the pectoral muscle, which is often an essential preprocessing step. Table 1 is systematically organized into columns detailing the methodologies, findings, and key contributions of each study, thereby offering an analysis of the various techniques employed for pectoral muscle segmentation in mammographic images. By synthesizing this information, Table 1 serves as an invaluable resource for researchers and practitioners alike, offering insights into the state-of-the-art approaches, challenges faced, and the effectiveness of different strategies in enhancing the accuracy and efficiency of segmentation processes. Furthermore, it is important to emphasize that methods for the segmentation of the pectoral muscle are currently still under study. The goal is to generalize the problem without regard to the characteristics of the incoming mammography to the system, especially in the search for tumors, masses, or microcalcifications. This focus aims to ensure that diagnostic processes are robust and universally applicable, regardless of the variability in mammographic images.

Table 1 demonstrates that while previous research has made great progress in pectoral muscle segmentation using a variety of CNN architectures and methodologies, there remains a distinct opportunity for innovation, particularly in addressing class imbalance and enhancing segmentation accuracy. Our proposed work leverages the advanced DeepLab v3+ architecture with a ResNet-50 backbone, introducing a class-weighted loss function to tackle the class imbalance issue effectively. Additionally, by employing comprehensive validation metrics and innovative data augmentation, alongside pre-segmentation analysis, our approach not only surpasses existing methods in accuracy but also in the reliability and clinical applicability of segmentation results.

It is imperative to highlight that the advancements documented in the literature are designed to enrich understanding of early detection strategies for breast cancer, utilizing non-invasive and expedient methodologies. This is particularly true in the context of medical imaging, which stands out as a swift and cost-effective approach. The importance of early diagnosis in breast cancer cannot be overstated, as it significantly impacts survival rates and treatment effectiveness. When breast cancer is detected in its localized stage, the 5-year relative survival rate is 99%, highlighting the critical role of early detection techniques such as mammography [3]. Mammograms play a vital role in identifying key indicators of breast cancer, including calcifications, masses, and architectural distortions, which can signify the presence of cancer at an early, more treatable stage. Calcifications, particularly microcalcifications, may suggest early breast cancer developments when found in certain patterns. Masses observed in mammograms, depending on their characteristics, can also indicate cancerous tissues and necessitate further diagnostic procedures. The detection of architectural distortions, despite being challenging, is crucial for identifying cancer that does not present as a distinct mass or calcification [33,34,35,36,37,38,39,40].

One of the key advantages of AI in breast cancer screening is its ability to work alongside radiologists to improve detection rates while reducing the workload on healthcare professionals. Studies have shown that AI, when combined with the expertise of radiologists, can outperform either one alone, detecting breast cancer more accurately and with fewer false positives, demonstrating a 2.6% improvement in detecting breast cancer over radiologists working alone and efficiently reducing unnecessary reviews of scans deemed “confident normal” [27,41]. Given these statistics and the continuous improvements in diagnostic technologies, there is a significant opportunity for research into novel methodologies for early breast cancer detection. This paper presents a method for the segmentation of the pectoral muscle based on CNN architecture with the DeepLab v3+ and a ResNet-50 backbone, aiming to further enhance the accuracy of mammographic image segmentation. By addressing challenges such as class imbalance with a class-weighted loss function and employing comprehensive validation metrics, our approach seeks to improve upon existing methods in detecting early signs of breast cancer, ultimately contributing to the ongoing efforts to increase survival rates and treatment success.

## 2. Materials and Methods

**Figure 1:**
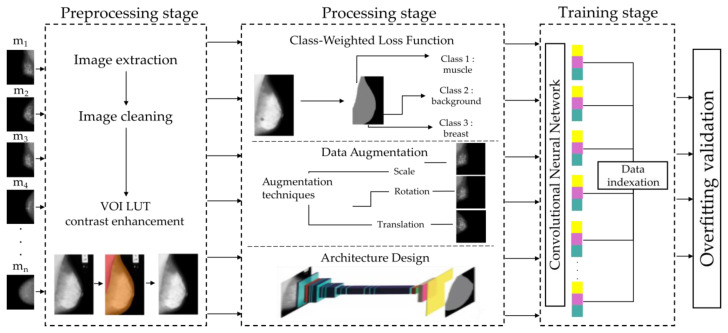
This illustration outlines the comprehensive procedure for classifying three distinct classes—pectoralis muscle, breast tissue, and background—with the primary goal of segmenting breast tissue by excluding the pectoral muscle. The process pipeline depicts several stages: initially, a preprocessing stage reduces data to the three target classes; this is followed by the implementation of a class-imbalance function to ensure equitable model training. To prevent overfitting, data augmentation techniques are employed. Subsequently, the pipeline involves a meticulously designed architecture. The concluding stages highlighted are the training of the model and the subsequent validation of its performance.

In this section, we describe the characteristics of the database and the features of the CNN, along with other considerations addressing the fundamental issue of how the pectoral muscle represents an interference in the detection of distortions, masses, and microcalcifications. It provides a comprehensive analysis of the database attributes, detailing the type and nature of the data, and the specific parameters and architecture of the CNN employed. Figure 1 provides an overview schematic of the process flow, illustrating the stages that will be described in this section.

### 2.1. Dataset Characteristics

The Mammographic Image Analysis Society (MIAS) dataset is an essential resource in the field of mammographic research, consisting of 322 scanned mediolateral oblique (MLO) images with a resolution of 1024 × 1024 pixels. Distributed by the UK National Breast Screening Programme, this dataset is renowned for its detailed annotations and diagnostic information [42]. The dataset contains diagnostic information, including biopsy results which classify findings into benign and malignant classes, and information on breast density, categorized as fatty, fatty-glandular, and dense-glandular.

Each MLO image has been segmented by a radiologist with two years of experience. This expert segmentation involves the accurate identification and outlining of relevant anatomical features of the pectoral muscle. The involvement of an experienced radiologist ensures a high level of accuracy and clinical relevance, providing a reliable ground truth for training and validating machine learning models, particularly for tasks like semantic segmentation.

### 2.2. Mammography Preprocessing

Digital mammography, through specialized X-ray equipment, is the gold standard imaging technique employed for the identification and assessment of breast cancer. This tomography technique allows radiologists to examine tissues for structural and morphological anomalies. The observed abnormalities are systematically assessed and categorized according to the BI-RADS standard, providing a standardized framework for the comprehensive evaluation of breast health. Specifically, digital images can be enhanced, modified, and magnified to assist accurate interpretation and in-depth research. Typically, digital mammography uses digital imaging and communications in medicine (DICOM), a standardized format for medical images that facilitates the seamless exchange of both images and patient information.

**Figure 2:**
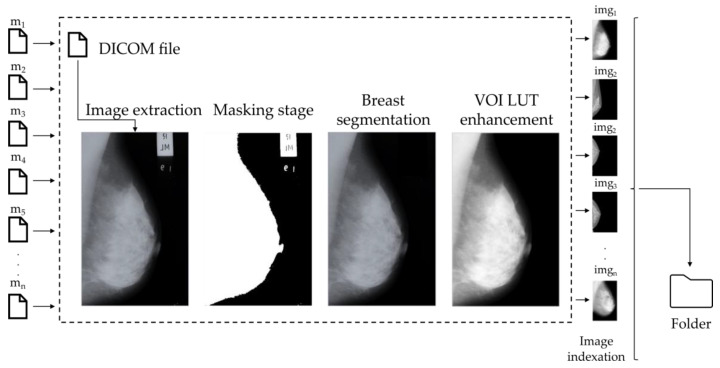
Image preprocessing pipeline consists of image extraction from the DICOM file, masking, breast segmentation, and contrast enhancement using the value of interest look-up table (VOI LUT).

In the current study, we have employed the Pydicom library for the processing and analysis of mammographic images. This Python-based tool is specifically engineered for the handling of DICOM files, which are pivotal for the comprehensive management of both imaging data and associated metadata. Our methodology employs image preprocessing techniques, including adaptive histogram equalization through VOI LUT and masking though threshold for segmentation processes [43]. However, the diverse standards of equipment can lead to challenges in accurately describing statistical features associated with spatial characteristics in mammographic images. To address these issues, transformations were applied to the radiography images in terms of segmentation and contrast enhancement, as depicted in Figure 2. These transformations were carried out to improve the quality and visual clarity of the images, allowing for more accurate analysis.

Breast segmentation specifically refers to the removal of commonly present annotations on the mammograms. These annotations typically consist of radiologist annotations or markings from the mammography equipment itself. The annotations in the mammograms can potentially interfere with the detection of different classes based on the intensity variations representing different tissues. The objective is to minimize a class that is regionally distant from the breast tissue. This involves identifying and segregating non-target areas, such as the pectoral muscle or background noise, which might otherwise distort the analysis due to their distinct intensity profiles compared to the breast tissue. By focusing on reducing the impact of these distant classes, the approach aims to enhance the accuracy of segmentation and classification of relevant breast tissue features. To achieve this, the segmentation concept involved isolating the largest region and removing the smaller regions corresponding to the labels. This process ensures that only the breast area is retained for further analysis, eliminating any sources of interference.

The segmentation process of the breast was conducted with the premise that the region of interest (ROI), which in this case is the breast, represents the largest area when compared to other annotations and the background. This approach assumes that the breast, being the primary focus of the image, occupies a significantly larger area than other elements. To achieve segmentation, the following steps were implemented.

The image was initially binarized, a process that transforms the original image into a binary image. This binary image comprises only two-pixel values, typically 0 (black) representing the background and 1 (white) representing the foreground or objects of interest. Let *I* be the original image, and *B(I)* represent the binarized version of *I*. The binarization can be expressed as a function *f* applied to each to each pixel *p* in *I*, as in Equation (1),

(1) B(I)={f(p)|p∈I}

where *f*(*p*) = 1 if *p* belongs to the foreground (breast) and *f*(*p*) = 0 otherwise. The binary image was then processed to identify distinct ‘islands’ or connected components. This was accomplished using the 4-connectivity technique, which considers pixels to be connected if they share an edge. The technique is based on the principle that two pixels are connected if they are both on and are neighbors horizontally or vertically. The set of islands in *B*(*I*) is given by {*I*_1_, *I*_2_,…*I_n_*}, where each *I_k_* is a connected component identified using the 4-connectivity technique. The segmentation of the breast, denoted as *S*, is defined in Equation (2). Among the detected islands, the largest one, naturally corresponding to the breast, was identified and isolated for further analysis. The largest detected island corresponding to the breast, was identified and subsequently isolated.

(2) $$S={argmax}_{I_{k}}\{area(I_{k})\}$$

where *area*(*I_k_*) represents the area of the island *I_k_*, and *argmax* function selects the island with the maximum area, which corresponds to the breast.

In our study, the value of interest (VOI) is defined to ensure that our mammographic analysis is focused on the breast tissue regions most likely to display early signs of pathology. This area is identified using a combination of anatomical landmarks and detailed analysis of breast tissue density profiles retrieved for each individual mammogram file. The selection of the VOI is predefined by the standards outlined in the BIRADS atlas. This standardization ensures that the VOI is not only specific to each patient’s unique breast anatomy but also consistent across all analyses, facilitating reliable and reproducible diagnostic assessments. To enhance the diagnostic visibility within this selected VOI, we apply a look-up table (LUT). This LUT is strategically designed to adjust the original pixel values to new values that enhance the overall image contrast. By mapping the pixel values, the LUT helps to bring out details in the mammographic images, thereby improving the tissue contrast through the mammogram [44].

This process restructured the classification of the images into three primary classes—muscle, breast tissue, and background—by removing the fourth class of annotations (which could be machine-generated labels or doctor’s notes). The removal of annotations from mammographic images is carefully managed to ensure no loss of critical diagnostic information. While these annotations are useful for clinical assessments, they do not contribute to the automated computational analysis techniques employed in our study, which focus solely on the anatomical and morphological features present in the images. To preserve all clinically relevant information typically contained in annotations, we meticulously document and store these data in accompanying metadata files. These files, formatted in accessible standards such as TXT or CSV, include comprehensive diagnostic details that complement the visual data, ensuring that no valuable information is excluded from the analysis process.

This strategic reduction in classes enhances the focus of the analysis on the anatomical and pathological features of the mammogram, crucial for the detection and assessment of breast cancer. Moreover, this preprocessing step can be regarded as a form of dimensionality reduction in image processing. By removing annotations, we reduce the complexity of the data while preserving the essential information relevant to breast cancer diagnosis. This reduction aids in minimizing potential distractions and noise in the data, enabling more efficient and accurate processing by the CNNs used in our study. Subsequently, once the images are free from interference, the process of eliminating the pectoral muscle regions was initiated. These regions were targeted for removal due to their potential to introduce interference into the images, primarily because of their intensity characteristics. The details of this process will be expounded upon in Section 2.3.

### 2.3. CNN Architecture and Parameterization for Semantic Segmentation

This section outlines the methodology for segmenting the three classes (i.e., pectoral muscle, breast tissue, and background) in such a way that allows for the distinction of different classes and the extraction of only the breast tissue class. The DeepLab v3+ architecture was employed in this study, integrated with a ResNet-50 backbone that was initially pre-trained on the expansive ImageNet dataset [45]. This integration was strategically chosen to optimize the segmentation stage. The decision to use a pre-trained model like ResNet-50 serves as the foundation for robust feature extraction. This approach significantly mitigates the need for a massive, task-specific dataset. Notably, ResNet-50 is distinguished by its depth and residual structures, which counter network degradation and gradient disappearance, challenges commonly encountered in traditional deep neural networks such as AlexNet or VGG-16. The ResNet-50 architecture is composed of 49 convolutional layers coupled with one fully connected layer (a pixel classification layer for this work), establishing a 50-layer network adept at preserving original information while efficiently managing network parameters [18,46,47].

**Figure 3:**
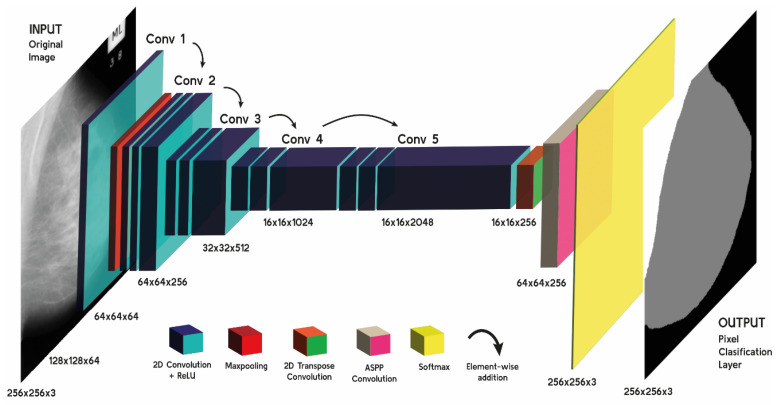
Hierarchical structure of the ResNet-50 CNN’s convolutional blocks. The initial layers apply a 7 × 7 convolution with 64 filters, stride 2, and a subsequent 3 × 3 max pooling with stride 2, serving as the entry point for down-sampling. This is followed by four main convolutional stages, denoted as conv2 to conv5, where each stage comprises multiple residual blocks with 3 × 3 convolutions. Specifically, conv2 is repeated 3 times, conv3 4 times, conv4 6 times, and conv5 3 times, progressively increasing the filter count from 64 to 2048. Each residual block within these stages includes a bottleneck design with 1 × 1 convolutions to reduce and then increase dimensions, optimizing computational efficiency. The architecture enables comprehensive feature extraction at various scales, crucial for accurate image segmentation tasks.

The adaptation of the pre-trained ResNet-50 to our specific context was achieved through fine-tuning. This process involved adjusting the CNN parameters using a subset of our specific dataset, ensuring the model’s relevance and applicability to mammographic image analysis. Fine-tuning allows the pre-trained model to maintain its existing feature recognition capabilities while aligning it more closely with the nuances of mammographic imaging [46]. A critical component of the DeepLab v3+ architecture is the atrous spatial pyramid pooling (ASPP) module. This module is essential for multi-scale analysis, a crucial aspect when dealing with mammographic images where anomalies can vary significantly in size and appearance. Furthermore, the model incorporates a decoder module that effectively merges the deep, semantic information from the ASPP with shallower, more detailed features. This leads to enhanced spatial accuracy in segmentation, a vital attribute for detailed medical analysis. The model terminates the process through a SoftMax layer, delivering pixel-level classification that is essential for precise segmentation in mammographic images [18,19,45]. The design of the ResNet-50 CNN, detailed in Figure 3, showcases the layered approach to convolutional processing, with each stage contributing to the model’s powerful image analysis capabilities.

### 2.4. Formulation of a Class-Weighted Loss Function for Enhanced Model Performance

Our work outlines a methodology for the automatic segmentation of the pectora muscle. However, a notable challenge in this study is the class imbalance issue, where we have three distinct classes: muscle, breast, and background. Notably, the background occupies a larger area compared to the breast, which in turn is larger than the muscle in terms of area coverage. This disparity in class sizes can lead to a biased learning process, where machine learning models, specifically in CNNs, might become inclined towards the more dominant classes (such as the background), potentially undermining the detection and segmentation of the less represented, yet clinically critical, classes like the pectoral muscle. In the semantic segmentation framework presented, let W represent the parameter matrix of the ResNet-50 architecture. The objective function, designed to train the segmentation model, is formulated as a combination of two distinct loss functions, given by Equation (3),

(3) $$L\left(W\right)=L_{class}\left(X_{class},W\right)+L_{seg}\left(X_{seg},W\right)$$

where $L_{class}$ and $L_{seg}$ denote the classification loss and segmentation loss functions, respectively, $X_{class}$ corresponds to the feature maps associated with the image classification task, and $X_{seg}$ pertains to the feature maps for pixel-wise segmentation. These functions are evaluated over the entirety of the training set, comprising both the image data and the corresponding ground truth segmentation masks.

To address the prevalent issue of class imbalance in segmentation—a scenario where most pixels often represent the background (non-segmented regions)—we have incorporated a weighted cross-entropy loss mechanism into our model. This was achieved by computing class weights from the training data, which were then seamlessly integrated into the pixel classification layer of the network. This ensured that each class was equally represented during the learning process, enhancing the model’s accuracy in segmenting each class. This approach is relevant in balancing the training process, ensuring that both segmented and non-segmented regions contribute appropriately to the model’s learning. The loss for each pixel $x_{i}\;$in relation to its ground truth label $y_{i}$ is computed through Equation (4) [19],

(4) $$L\left\{\begin{array}{ll} \alpha \cdot \log\left(1-\sigma \left(x_{i}\right)\right) & \;\;\;\;if\;y_{i}=0\\ \beta \cdot \log\left(\sigma \left(x_{i}\right)\right) & \;\;\;\;if\;y_{i}=1 \end{array}\right.$$

where σ is the sigmoid activation function to convert the network output into a probability distribution. The weights α and β are determined based on the relative frequency of segmented and non-segmented pixels in the training set, given by Equations (5) and (6),

(5) $$\alpha =\lambda \cdot \frac{\left|Y^{+}\right|}{\left|Y^{+}\right|+\left|Y^{-}\right|}$$

(6) $$\beta =\frac{\left|Y^{-}\right|}{\left|Y^{+}\right|+\left|Y^{-}\right|}$$

where $\left|Y^{+}\right|$ and $\left|Y^{-}\right|$ denote the counts of pixels within and outside the segmented regions, respectively. The parameter λ serves as a tuning factor, providing an additional degree of control to mitigate the bias towards the more prevalent class. This formulation of the loss function ensures that the ResNet-50 model is not only accurately segmenting mammographic images but also maintaining a balanced sensitivity to all crucial regions within these images.

### 2.5. CNN Training for Semantic Target Regions Segmentation

As part of the training procedure, it is crucial to have a ground truth reference to accurately identify regions that truly belong to a specific class. As described in Section 2.1, Dataset Characteristics, the labels were manually annotated by a radiologist. The inclusion of these specific labels enabled our model to accurately distinguish between critical regions in the mammographic images, which was crucial for the effective training and subsequent validation of the model in identifying and analyzing various breast tissues in the images. We applied random affine transformations, including translations of up to 10 pixels in any direction and rotations within a 30-degree range.

The application of three key image transformations—scale, rotation, and translation—has augmented our dataset to 1288 images. The augmentation is designed with a dual purpose: firstly, to increase the volume of data available for model training, and secondly, to introduce a degree of variability that mirrors fluctuations encountered in clinical environments. Such variations may arise from different patient positioning, the diversity of breast sizes and shapes, and the variable imaging parameters typically used in medical practice. By enriching the dataset with these augmented images, the robustness of our machine learning model is significantly enhanced. It gains the capability to recognize and accurately interpret mammographic images that may otherwise deviate from the ‘standard’ presentations due to these real-world variations. This strategic approach to dataset augmentation is critical for ensuring that the model is not only well-trained on a sizeable and varied dataset but also adept at handling the unpredictability and diversity of clinical images. Consequently, this reinforces the model’s diagnostic accuracy, making it a more reliable tool for radiologists and clinicians in the early detection and diagnosis of breast cancer in patients [43,44,46].

Our study utilizes a dataset consisting of 1288 mediolateral oblique (MLO) mammograms, standardized to a resolution of 256 × 256 pixels. This dataset is meticulously divided into 70% for training (902 images), 20% for validation (258 images), and 10% for testing (128 images), adhering to the best practices in medical image analysis. Such a comprehensive dataset is crucial for developing robust machine learning models that demonstrate high generalizability across various diagnostic scenarios.

The strategic division of the dataset into training, validation, and testing segments is designed to optimize the performance and generalizability of the models. This segmentation approach is supported by evidence in the literature, which underscores the importance of substantial datasets in enhancing the accuracy and reliability of diagnostic tools. For instance, extensive datasets have been shown to improve segmentation accuracy significantly, as noted by [17], who emphasized the benefits of large training sets for the precision of models in clinical applications. Additionally, the diversity in training and validation sets, as discussed by [18], ensures that the models remain adaptable and effective under varying imaging conditions [18,19,47].

Stochastic gradient descent with momentum (SGDM) was used for training the semantic segmentation CNN, which is particularly effective for handling patterns in mammographic images. The SGDM algorithm is given by the velocity update rule expressed in Equation (7) and the weight update rule presented in Equation (8),

(7) $$v_{\left\{t+1\right\}}=\mu \cdot v_{t}-\eta \cdot \nabla L\left(W_{t}\right)$$

(8) $$W_{t+1}=W_{t}+v_{t+1}$$

where $v_{t}$ represents the velocity at the current iteration t, and$\;v_{\left\{t+1\right\}}$ is the velocity at the next iteration. The parameter μ is the momentum factor, which helps in maintaining the direction and speed of the optimizer, making it less susceptible to local minima. The learning rate η was set to 0.001, allowing for gradual adjustments to the model weights without overshooting. $\nabla L\left(W_{t}\right)$ denotes the gradient of the loss function with respect to the weights W at iteration t. This method updates the process of reaching the global minimum of the loss function in a more uniform way. By employing SGDM with a calibrated learning rate and momentum—50 epochs and a mini-batch size of 2—we achieved an accurate segmentation in mammographic images.

### 2.6. Validation and Performance Assessment of Segmentation Models

A validation was carried out. Each label—background, breast tissue, and pectoral muscle—was evaluated independently, providing a detailed assessment of the segmentation’s precision for each anatomical feature.

The Dice coefficient evaluates the similarity of two regions, specifically comparing the predicted segmentation against the ground truth for each label independently, which is sensitive to structures. For a given label, the Dice coefficient D is calculated in Equation (9),

(9) $$D{\left(P,G\right)}_{k}=\frac{2\times \left|P_{k}\cap G_{k}\right|}{\left|P\right|+\left|G\right|}$$

where $\left|P_{k}\right|$ represents the predicted set of pixels for a label k, $\left|G_{k}\right|$ is the number of pixels belonging to label k in the ground truth set, and $\left|P_{k}\cap G_{k}\right|$ is the count of pixels common to both.

On the other hand, the Jaccard index computes the Intersection over Union, an approach that is particularly suitable when the ROI varies in scale. This index is computed for each label, designated as k, within an image. This computation yields three Jaccard scores, each evaluating the segmentation accuracy by considering both the correctly predicted pixels and the overall size of the predicted and actual regions, as detailed in Equation (10):

(10) $$J{\left(P,G\right)}_{k}=\frac{\left|P_{k}\cap G_{k}\right|}{\left|P_{k}\cap G_{k}\right|}$$

Lastly, the confusion matrix was computed for quantifying classification accuracy. Constructed as an n×n grid, where n indicates the number of distinct labels, the matrix delineates the pixel-wise accuracy of segmentation predictions. Entries $C_{ij}$ within the matrix enumerate the pixels of the true class i that the model erroneously predicted as class j. Diagonal elements reflect the model’s accurate predictions, affirming its competence in identifying the respective classes, while off-diagonal elements provide a perspective of the model’s misclassifications failures.

The following section presents the results achieved in the classification of regions, along with the validation metrics that provide evidence of the promising outcomes of the methods employed.

## 3. Results

### 3.1. Training Dynamics and Initial Performance Evaluation of the CNN Model

**Figure 4:**
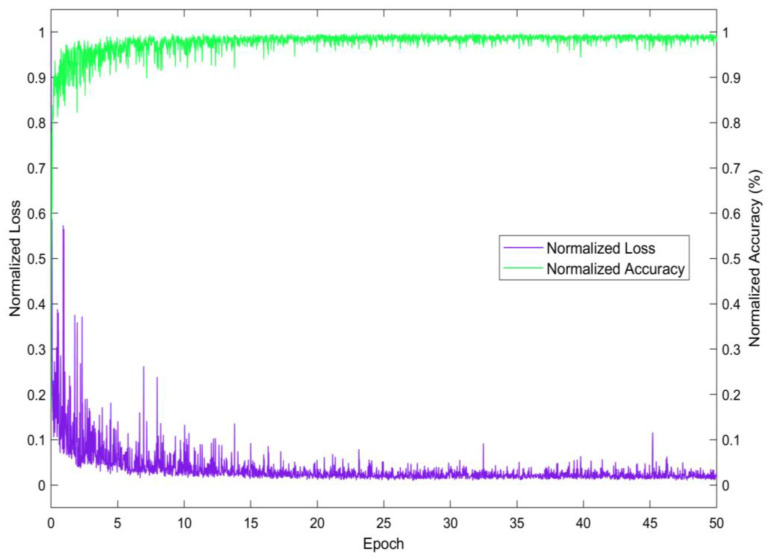
Training dynamics of the CNN over 50 epochs, displaying normalized loss (purple) and normalized accuracy (green). The declining loss and stabilizing accuracy signify model optimization, despite minor fluctuations indicating the complexity of the segmentation task.

In the results section of our study, we present the analysis of the performance of the CNN model, and the progression of training over time. Figure 3 provides a representation of the model’s learning dynamics across 50 epochs, capturing both the normalized loss and accuracy. As depicted in Figure 4, the normalized loss shown in purple exhibits a sharp decline in the initial epochs, indicating rapid learning and improvement in the model’s ability to segment the mammographic images accurately. This initial phase is characterized by adjustments to the model’s weights and biases, reflecting the optimization process of the CNN. Simultaneously, the normalized accuracy, depicted in green, shows a steady ascent towards stabilization. This trend demonstrates the model’s increasing proficiency in correctly classifying each pixel into one of the three predefined classes: pectoral muscle, breast tissue, and background. Additionally, the data offer a view of learning efficacy, evidenced by the decreasing trend of the loss towards a minimal level and the stabilization of accuracy at a high point, indicative of an effective training. The variance in loss and sporadic peaks in later training stages highlight the complexities of the segmentation task, yet the general direction suggests a resilient and finely adjusted model.

### 3.2. Evaluation of Model Segmentation Performance

The Dice coefficient and the Jaccard index were employed as the primary metrics to assess the model’s performance across a dataset of 322 images, each annotated with three distinct labels that correspond to pectoral muscle, breast, and background. The spatial overlap between the expected segmentation and the radiologist’s ground truth is used by the Dice coefficient to assess the method precision. For every image, the coefficient was calculated for every label. The Dice coefficient, as a measure of accuracy for image segmentation, compares the spatial overlap between model-predicted segmentations and the ground truth. Values approaching 1 signify near-perfect overlap, a hallmark of precise segmentation.

**Figure 5:**
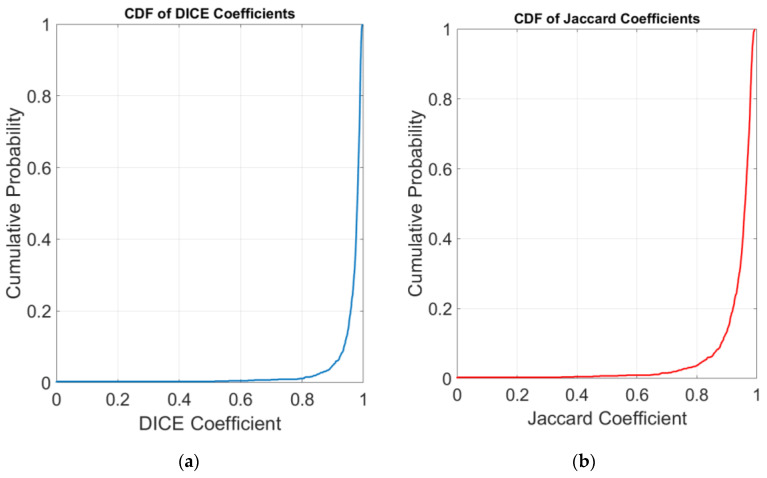
CDF plots for (**a**) Dice coefficients and (**b**) Jaccard coefficients. The plots illustrate the proportion of images within the dataset that fall below or at specific coefficient values, indicating the model performance in segmentation tasks. A steep rise to the upper limit of 1 on both plots signifies that most images achieve a high degree of segmentation accuracy, with the Dice coefficient plot demonstrating a slightly earlier convergence, suggesting an overall higher segmentation success rate across the dataset as compared to the Jaccard index.

Figure 5 presents a cumulative distribution function (CDF) analysis for both the Dice coefficient and the Jaccard index, serving as critical measures of segmentation performance for the three classes. Figure 5a shows a steep curve reaching towards 1 for the Dice coefficient, suggesting that most of the segmented images closely match the ground truth performed by the radiologist. This pattern denotes a highly successful segmentation output, as the Dice coefficient evaluates the overlap between the predicted and true segmentation areas. Figure 5b presents the CDF for the Jaccard index, which mirrors the pattern observed in the Dice coefficient yet provides a distinct perspective by measuring the ratio of the Intersection over Union (IoU) of the segmentations. The Jaccard index is particularly informative in describing the balance between precision and recall within the model performance. The results indicate a substantial proportion of images with high index values (near to 1), thus confirming the model efficacy.

**Figure 6:**
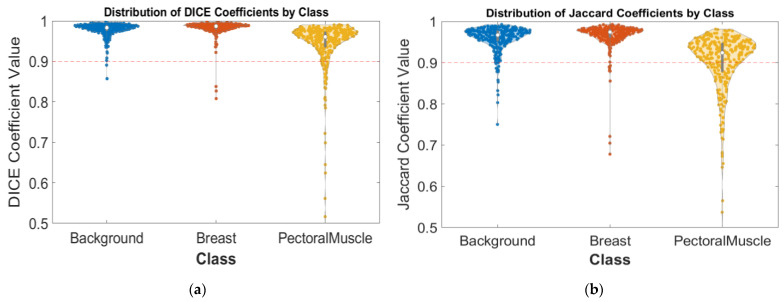
(**a**) Distribution of Dice coefficients by class. This graph illustrates the spread of Dice coefficient values across the three classes: background (blue), breast (orange), and pectoral muscle (yellow). Median values are indicated by dashed lines, with the median for background at 0.98, breast at 0.99, and pectoral muscle at 0.96, highlighting the model precision in segmenting these classes; (**b**): Distribution of Jaccard coefficients by class. Like the Dice coefficient distribution, this graph displays the spread of Jaccard coefficient values for the same classes. The medians, marked by dashed lines, show a high level of accuracy with 0.97 for both background and breast, and 0.92 for pectoral muscle, underscoring the model effective segmentation ability.

Figure 6a provides a representation of the distribution of Dice coefficients for each class within the dataset: background, breast, and pectoral muscle. It showcases the variability, and the central tendency of the Dice coefficient values across different segmentation tasks performed by the model. The median Dice coefficient for the background class is 0.98, indicating that the model is adept at accurately segmenting the background from the primary regions of interest. The breast class shows a median of 0.99, and lastly, the pectoral muscle class presents a median of 0.96, which, while slightly lower than the breast tissue, still represents an acceptable level of accuracy in segmentation. This confirms that the model maintains strong performance even when segmenting more challenging and intricate anatomical structures. Figure 6b showcases the distribution of Jaccard coefficients across the three segmented classes. The median values for the background and breast classes are both 0.97, which suggests a strong overlap between the model predictions and the ground truth annotations for these categories. For the pectoral muscle class, the median is slightly lower, at 0.92, which still denotes an acceptable degree of accuracy.

**Figure 7:**
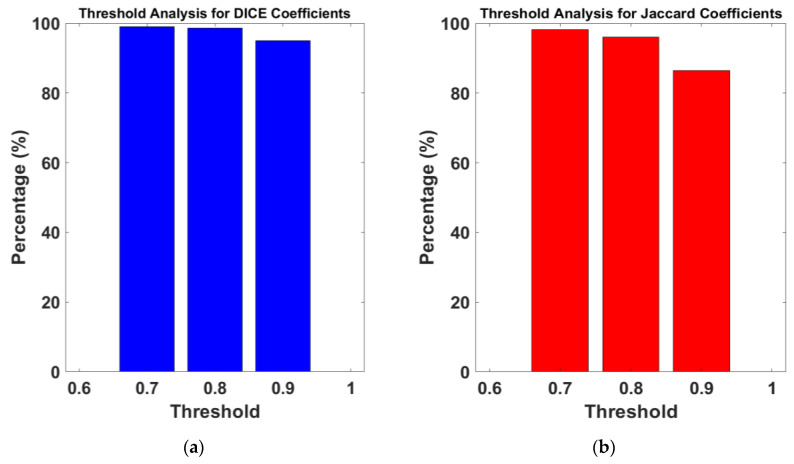
Threshold analysis for Dice and Jaccard coefficients. The histograms represent the percentage of images exceeding specified threshold values for (**a**) Dice coefficients and (**b**) Jaccard coefficients. A high percentage of images surpass the threshold at each incremental level, demonstrating the model effectiveness in segmentation tasks across the dataset.

Figure 7 presents a threshold analysis (Figure 7a for the Dice coefficients and Figure 7b for Jaccard coefficients), reflecting the proportion of images that meet or exceed various threshold levels in segmentation performance. For the Dice coefficients, the histogram shows that an overwhelmingly large percentage of images exceed the 0.6 threshold, with the percentage remaining consistently high even as the threshold increases. Notably, there is only a slight decrease in the percentage of images meeting the thresholds as they approach the perfect overlap score of 1, highlighting the model robustness for discernment within classes. Similarly, the histogram for the Jaccard coefficients indicates that a significant majority of images surpass the lower thresholds, and a high percentage of images maintain this standard as the threshold levels increase. This trend is indicative of the model precision and reliability in segmenting the images, ensuring a consistent performance even when stringent criteria are applied.

Table 2 presents a comparison between the areas labeled by the CNN and those delineated by the ground truth. The breast class showcases a high degree of concordance, with the CNN labeled area slightly overestimating compared to the radiologist assessment. This is reflected in the high Jaccard and Dice scores, indicating a strong overlap and similarity.

**Figure 8:**
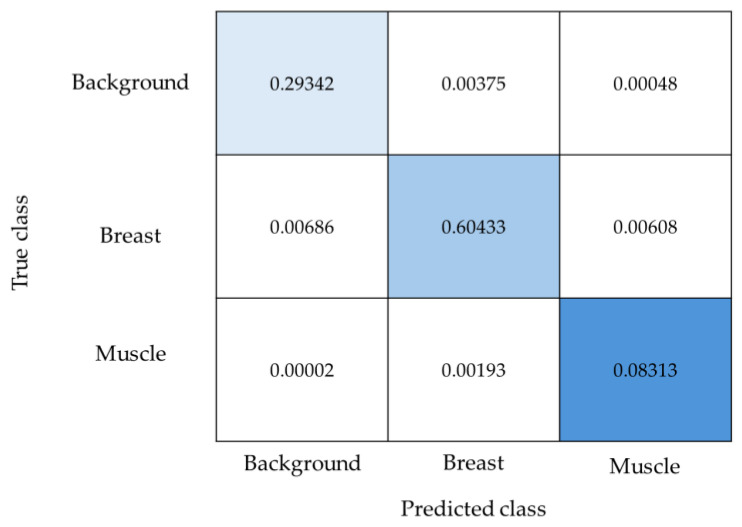
This normalized confusion matrix illustrates the distribution of predictions across the classes of background, breast, and muscle. The intensity of the blue color correlates with the normalized frequency of predictions for each class, with the numeric values indicating the proportion of the total predictions.

Figure 8 presents the normalized confusion matrix for a three-class classification problem. The confusion matrix is a tool in evaluating the performance of a classification model, providing insights into how well the model is identifying each class. The results exhibit a strong diagonal trend indicative of high classification accuracy. The most prominent value on the diagonal is 0.60433, attributed to the breast class, which reflects the model in identifying this class correctly. The value of 0.08313, corresponding to the muscle class, represents the smallest normalized value in the confusion matrix. It indicates a smaller proportion of muscle class predictions, which could be due to fewer instances of this class within the dataset or a more challenging classification task for the model in distinguishing muscle from other classes. Together, the values on the diagonal sum to 0.98, which underlines the overall model accuracy and reliability. This high cumulative value on the diagonal signifies that the model has achieved a substantial degree of correct classifications across all classes.

A critical component of our methodology was the employment of a ground truth mask. This benchmarking tool was instrumental in calibrating the accuracy of the CNN’s segmentation outputs, thus validating the model’s operational integrity. Data processing constituted a significant aspect of our research. Advanced techniques, notably data augmentation, were employed to fortify the model’s robustness and adaptability. The model underwent extensive training, spanning 1411 min over 50 epochs. This rigorous training regimen was indispensable in achieving the model’s laudable final validation accuracy of 97.77%. The post-processing of segmented labels was a crucial phase, ensuring the refinement and utility of the resultant data. The tabulated data further elucidates the model’s performance across various classifications. Notably, in the Breast category, the model exhibited exceptional precision, evidenced by an average Dice coefficient of 0.982550 and a Jaccard index of 0.966275. Although accuracy metrics in other categories like Background and Pectoral Muscle were slightly lower, they nonetheless highlight potential avenues for future optimization.

**Figure 9:**
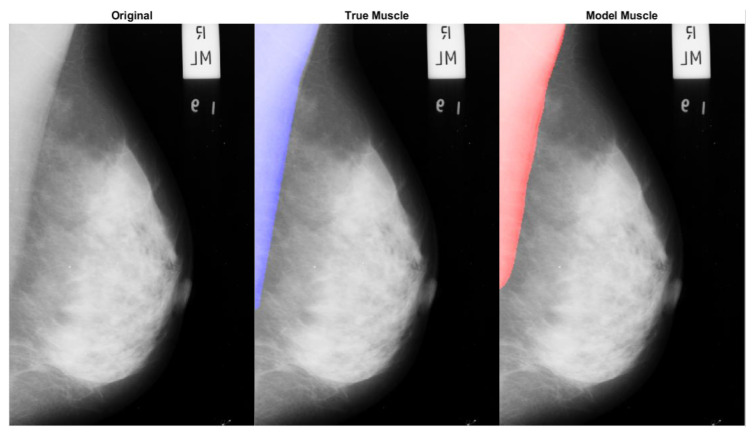
Comparative visualization of mammogram segmentation, featuring the original image alongside the ground truth and automated model segmentations of the pectoral muscle. The original mammogram is displayed on the left, with the ground truth segmentation overlaid in blue at the center, and the model’s segmentation is overlaid in red on the right. This figure illustrates the model’s capability to identify the pectoral muscle against the expert-delineated ground truth, providing a concise evaluation of segmentation accuracy.

Figure 9 presents a comparative visualization of the original mammographic image, the ground truth muscle segmentation, and the muscle segmentation as identified by our CNN model. In the ‘True Muscle’ panel, the blue overlay indicates the expert-annotated region delineating the true muscle area, serving as a reference standard for segmentation. The ‘Model Muscle’ presents a red overlay which represents the muscle region as segmented by our CNN model, demonstrating the model’s ability to closely replicate the true segmentation.

## 4. Discussion

One of the current issues in detecting structural anomalies in mammograms based on Computer Vision and AI algorithms is the class imbalance when studying specific anatomical regions of the breast. In this regard, it is necessary to precisely delimit the ROIs, i.e., the breast regions, so that the learning models only analyze these regions and there are no tissues that could be considered as interference. Despite advances in pectoral muscle segmentation, disparities in accuracy among different classes, particularly the lower metrics observed for pectoral muscle classification, are still challenges within the field of mammography analysis. Therefore, this study aimed to address the class imbalance problem in such a way that all three classes could be segmented using a CNN, with the aim of increasing segmentation performance compared to the state of the art. However, one of the challenges that is always present in supervised learning studies is the amount of information for training the network. In this regard, a solution was also proposed to address the problem of the lack of medical images by increasing the quantity of mammograms through scale transformation, rotation, and translation techniques. Data augmentation strategies have proven to be particularly effective, illustrating that a diverse and enriched dataset is essential for developing robust diagnostic tools in scenarios where mammograms are limited in quantity.

Our paper incorporates studies that employ common datasets such as MIAS, IN-BREAST, and DDSM, allowing for a direct evaluation of the performance metrics associated with each method. Table 3 summarizes the outcomes from multiple publications, detailing the segmentation techniques employed, datasets used, and performance metrics including Dice coefficients, and overall accuracy. These metrics are crucial for assessing the efficacy of each method in accurately delineating the pectoral muscle from breast tissue, which is a vital step in enhancing the diagnostic process for breast cancer. The quantitative data extracted from these studies indicate a progression in segmentation accuracy over recent years, reflecting advances in computational techniques and algorithmic design. For instance, the use of CNNs with skip connections has been shown to achieve high accuracy, as evidenced by the performance of [17]’s approach with a Dice coefficient of 96% and accuracy of 98%. This performance is contrasted against more traditional methods, which generally report lower metrics, illustrating the benefits of integrating advanced neural network architectures in medical image analysis. Furthermore, the analysis delves into the technical aspects of each technique, discussing how modifications in network architecture, such as the introduction of holistically nested edge detection in the work of [18], contribute to a higher Dice coefficient of 97.5%.

Our results lay on a framework for future studies, suggesting that models trained with even more diverse datasets and with specifically segmented breast regions could further improve outcomes for breast cancer findings based on mammograms. Future studies might also research the integration of clinical data to amplify predictive capabilities to individual patient profiles. The promise of enhancing data in screening breast cancer pre-stages, may have a full potential through iterative refinement and rigorous validation to ensure reliability and efficacy in clinical applications.

## 5. Conclusions

This paper presents a method for pectoral muscle segmentation in mammograms based on CNNs, applying a novel approach that includes a function to address the class imbalance problem in pectoral muscle, breast, and image background, along with data augmentation using translation, scaling, and rotation functions. This focus minimizes the interference from non-relevant tissues, thus enhancing the training of automated learning models. By employing a multi-layered CNN architecture, we have achieved a segmentation accuracy of 98.09%. The results are indicative of our proposal’s effectiveness in classifying breast tissue from mammographic images. The significant role of data augmentation in reinforcing the robustness of the model, along with 50 epochs for training, has achieved a final validation accuracy of 97.77%. For the validation stage, the model demonstrated an average Dice coefficient of 0.982550 and a Jaccard index of 0.966275. These metrics not only highlight our method’s potential to classify breast tissue but also provide clear directions for future enhancements. The performance in other categories such as the background and pectoral muscle, though slightly lower, has offered valuable insights into areas requiring further improvement. Overall, the outcomes of this study make significant contributions to the field of medical image processing, emphasizing the effectiveness of CNNs in the accurate segmentation of the pectoral muscle along with the class imbalance function.

## Figures and Tables

**Table 1:** Resume of the state-of-the-art in pectoral muscle segmentation from mammograms, presenting a comparative analysis across various studies.

Authors	Year	Dataset Used	Methodology	Main Findings	Challenges Addressed	Future Directions
Rampun et al. [18]	2019	MIAS, BCDR, INbreast, CBISDDSM	Modified HED network	Jaccar: 94.8 ± 8.5% and Dice: 97.5 ± 6.3%	Identification of indistinct boundaries from dense tissues	Refine architecture for enhanced detection
Soleimani and Michailovich [19]	2020	MIAS, CBIS-DDSM, INbreast	Deep learning and graph-based processing	Average DSC and ACC are 97.22% and 99.64%, respectively	Delineating pectoral muscle boundary	Integrate with CAD systems
Firdi et al. [27]	2022	Not specified	Edge detection and Hough transform for muscle removal	Accuracy: 68.67%, Precision: 64.71%, Specificity: 57.14%, Sensitivity: 80.49%	Removing pectoral muscle for cancer detection	Develop CNN for better analysis accuracy
Tiryaki and Kaplanoglu [28]	2022	DDSM	ResNet50-U-net with Tversky loss	Breast density classification accuracy reached 76.01%, aligning with the recent literature results	Accuracy in diverse breast tissues	Explore deep learning for tissue segmentation
Ranjbarzadeh et al. [29]	2023	Not specified	ME-CCNN with multi-encoded images	Proposed framework outperforms baselines on two datasets	Segmenting breast tumors	Refine encoding techniques and CNN models
Jacinta C. Anusionwu et al. [30]	2023	MIAS	Region-based standard Otsu technique	Average Jaccard = 93.2%, False Positive: 3.54% False Negative: 5.68%	The issue of biased results in mammographic analysis due to the presence of PM was addressed	Suggested the use of images obtained after removing PM for further analysis
Xiang Yu, Shui-Hua Wang, Yu-Dong Zhang [31]	2023	CBIS-DDSM and INbreast	Deeplabv3+ and a multiple-level thresholding.	Sensitivity of 0.87 at 2.86 FPI (False Positive rate per Image)	Detecting breast mass in mammography images with varied breast densities was addressed	Further exploration into refining the detection and segmentation methodology
Chen et al. [31]	2024	Joanne Knight Breast Health Cohort	Algorithm combining binarization and edge detection	Algorithm’s mean error is 12.22%, lower than Libra’s 20.44%	Pectoral muscle removal in MLO images	Enhance algorithm for diverse datasets
Rehna Kalam et al. [32]	2024	MIAS	Entropy-based Fuzzy C-Means Clustering and RMCNN	Achieved classification accuracy of 99.45%, outperforming existing methods with lower accuracies	Accurately characterizing and classifying breast cancer images from mammograms, particularly the removal of pectoral muscle	Further exploration into optimizing the methodology for even higher accuracy

**Table 2:** Results per class of training and testing of our proposed architecture on MIAS database.

Class	CNN Labeled Area [Pixels]	Radiologist Labeled Area [Pixels]	Average of Jaccard	Average of DICE	Absolute Error	Relative Error
Breast	119,523,049	118,119,172	0.97	0.98	1,403,877	0.012
Background	57,634,559	58,147,516	0.96	0.98	512,957	0.009
Pectoral Muscle	16,475,672	17,366,592	0.89	0.93	890,920	0.051

**Table 3:** Comparative analysis of pectoral muscle segmentation techniques.

Author	Year	Technique	Dataset	DICE	Accuracy	Class-Weighted Loss Used
Yanfeng Li et al. [25].	2013	Pectoral muscle segmentation using homogeneous texture	Mini-MIAS, DDSM	Not reported	90.06% (Mini-MIAS), 92.00% (DDSM)	No
Mario Mustra et al. [41].	2016	Review of segmentation methods for pectoral muscle in mammograms	MIAS, DDSM (varias bases de datos)	Not reported	Not reported	No
S. Michahial et al. [21].	2017	Algorithm for automatic seed point selection in breast ultrasound	160 breast ultrasound images	Not reported	87.00%	No
Andrik Rampun et al. [18].	2019	Modified holistically nested edge detection network	MIAS, INbreast, BCDR, CBIS-DDSM	97.5%	Not reported	No
Muhammad Junaid Ali et al. [17].	2020	Convolutional neural network with skip connections	MIAS, INBREAST, DDSM	96.00%	98.00%	No
Juanita Hernández López et al. [47]	2023	Fat removal preprocessing	BCDR, INbreast, private dataset	94.00%	94.18%	Yes
Fausto David Cortes-Rojas et al.	2024	Advanced CNN with class imbalance adjustment	MIAS, INBREAST	98.09%	97–77%	Yes

## Data Availability

All data used in this research are available upon request from the corresponding author.
